# Latent Profiles of Early Maladaptive Schemas and Their Associations With Suicide Risk Factors in Patients With Mood Disorders

**DOI:** 10.1002/cpp.70069

**Published:** 2025-03-29

**Authors:** Chanhee Park, C. Hyung Keun Park

**Affiliations:** ^1^ Asan Institute for Life Sciences Asan Medical Center Seoul Republic of Korea; ^2^ Department of Psychiatry Asan Medical Center Seoul Republic of Korea

**Keywords:** early maladaptive schemas, interpersonal theory of suicide, latent profile analysis, suicidal ideation, suicide risk

## Abstract

**Purpose:**

Although early maladaptive schemas (EMSs) have been linked to suicidal ideation, their heterogeneous nature has not been fully explored in this relationship. This study sought to identify distinct latent profiles based on 18 EMSs in a clinical sample and examine how these profiles differ in relation to suicidal ideation and suicide risk factors, such as perceived burdensomeness, thwarted belongingness and fearlessness about death.

**Method:**

Data from routine clinical assessments of 799 outpatients with mood disorders (aged 18–49 years, 513 female, 286 male, *M*_*age*_ = 28.71) were analysed. Latent profile analysis was performed to identify distinct EMS profiles, and their associations with suicide risk factors were examined using the Bolck–Croon–Hagenaars method.

**Results:**

Five distinct EMS profiles emerged: *Low*, *Below Average*, *Above Average*, *Specific Elevation* and *High Risk*. The *Specific Elevation* profile demonstrated specifically elevated levels of social isolation/alienation, defectiveness/shame, failure, dependence/incompetence and insufficient self‐control/self‐discipline, while the levels of most other schemas were similar to the *Above Average* profile. The *High Risk* profile showed elevated levels across all schemas. Both the *Specific Elevation* profile and the *High Risk* profile exhibited the highest levels of perceived burdensomeness, thwarted belongingness and suicidal ideation, with no significant differences between them. Fearlessness about death did not differ among the profiles.

**Conclusion:**

The identified EMS profiles offer unique utility in predicting perceived burdensomeness, thwarted belongingness and suicidal ideation. The specific schemas that showed elevation in the *Specific Elevation* profile may serve as promising targets for mitigating suicide risk in patients with mood disorders.

**Summary**

- Heterogeneous nature of EMS was examined.
- Five EMS profiles were identified by LPA.
- The *Specific Elevation* and *High Risk* profiles showed highest levels of suicide risk.
- The *Specific Elevation* profile had uniquely elevated levels of certain schemas.
- Targeting elevated schemas in the *Specific Elevation* profile may reduce suicide risk.

## 1 Introduction

Early maladaptive schemas (EMSs) are conceptualised as self‐perpetuating, relatively stable cognitive patterns that affect thought and behaviour in dysfunctional ways (Young et al. 2006). EMSs typically develop from unmet basic needs, adverse experiences during childhood and individual temperament (Beck 1991; Young et al. 2006). When triggered, EMSs dominate thoughts and feelings, leading to intense negative emotions and maladaptive coping behaviours (Beck 1991; Young et al. 2006). The concept of schemas in psychotherapy originates from Beck's cognitive theory (Beck 1970), which posited schemas as interacting factors with symbolic situations to elicit negative automatic thoughts, eventually leading to psychopathology. Building on this work, Young et al. (2006) expanded the concept by identifying 18 specific EMSs, which encompass a range of dysfunctional beliefs and expectations about oneself and relationships with others. EMSs have demonstrated high stability over time in depressed patients, even after controlling for the severity of depressive symptoms (Riso et al. 2006; Wang et al. 2010). This persistence underscores their potential role as enduring risk factors for various mental health problems, including suicidal behaviour.

Suicide is a major global public health concern, with annual mortality rates estimated to exceed 700,000 worldwide (World Health Organization 2023). It is important to recognise that completed suicide is not an isolated event, but rather the culmination of a progression through various stages (Klonsky et al. 2018). Prevailing theories in the field of suicidology generally concur that suicidal individuals initially develop suicidal ideation, influenced by various psychological risk factors, including perceived burdensomeness and thwarted belongingness (Klonsky et al. 2018; Van Orden et al. 2010). Consequently, it is crucial to identify the factors that significantly contribute to the development of these risk factors and suicidal ideation within clinical populations, especially considering that individuals with psychiatric disorders are particularly vulnerable to suicide risk (Moitra et al. 2021).

As schemas are theorised to be dysfunctional cognitive patterns (Young et al. 2006), it has also been proposed that suicidal ideation may represent a particular information processing pattern, stemming from a sense of entrapment and hopelessness (Flink et al. 2017; Joiner et al. 2012). Widely accepted theories in suicidology, such as the interpersonal theory of suicide (IPTS) (Chu et al. 2017; Van Orden et al. 2010), also identify cognitive risk factors for suicide that are conceptually associated with EMSs. The IPTS posits that individuals develop suicidal desire when experiencing perceived burdensomeness and thwarted belongingness (Joiner 2005), which align with defectiveness/shame and social isolation/alienation schemas, respectively (Pilkington et al. 2021). Similarly, hopelessness, a central factor in many suicide theories, conceptually parallels negativity/pessimism schemas (Pilkington et al. 2021). Empirical research has consistently demonstrated a positive association between elevated EMSs and suicidal behaviour across various conditions, including patients with mood disorders (Nilsson 2016), patients with obsessive‐compulsive disorder (Khosravani et al. 2017) and the general population (Milesi et al. 2023). It has also been demonstrated that EMSs are significantly associated with the risk of repetition of suicidal behaviour (Dale et al. 2010).

From a clinical perspective, identifying specific schemas most strongly associated with suicidal ideation is crucial for informing risk assessment and guiding the selection of therapeutic targets. However, despite the growing body of research on EMSs and suicide risk, our comprehension of how different schema profiles relate to suicide risk remains incomplete. A meta‐analysis examining the relationship between EMSs and suicidal ideation has suggested that EMSs may have a cumulative effect on suicide risk (Pilkington et al. 2021). This idea aligns with the schematic appraisal model of suicide (Johnson et al. 2008, 2010), which posits that a “cluster” of cognitive schemas could contribute to suicide. Yet, most studies have examined EMSs individually or in predefined domains, potentially overlooking complex schema combinations and interactions that could provide a more nuanced understanding of suicide risk.

To address this gap in our understanding, employing advanced statistical techniques such as latent profile analysis (LPA) could be beneficial (Pilkington et al. 2021). As a person‐centred approach, LPA allows for the identification of distinct profiles based on EMS patterns, potentially revealing how different configurations of schemas relate to suicide risk (Muthén and Muthén 2017). An LPA approach for investigating EMSs and suicidal ideation is congruent with the complex, multifaceted nature of both schema theory and suicidal behaviour. It acknowledges that individuals may have elevations across multiple schemas simultaneously and that these elevation patterns, rather than individual schemas alone, may be most informative in understanding suicide risk.

To summarise, while the relationship between EMSs and suicide risk has been established in the literature, there is a need for more nuanced, person‐centred approaches to understand this relationship. By employing LPA to examine how different EMS profiles relate to suicide risk factors, we can potentially identify high‐risk schema configurations, inform more targeted interventions and contribute to the refinement of existing suicidal behaviour theories. In this study, our primary objective was to identify latent patterns of 18 EMSs using LPA. In addition, we aimed to examine how the latent profiles differ across various suicide risk factors. Specifically, we sought to observe differences in suicidal ideation, as well as risk factors for suicide proposed by the IPTS, including perceived burdensomeness, thwarted belongingness and fearlessness about death.

## 2 Methods

### 2.1 Study Sample and Data Collection

We utilised data from assessments administered as part of the routine clinical practice for new patients at the Mood Disorder and Suicide Prevention Clinic within the Department of Psychiatry at Asan Medical Center, a tertiary hospital in Seoul, Republic of Korea. The study included 799 outpatients aged between 18 and 49 years (64.20% female, *M*_*age*_ = 28.71, *SD*_*age*_ = 8.51) who visited the clinic from February 2022 to May 2024, presenting with mood symptoms or suicide risk. Inclusion criteria encompassed a confirmed clinical diagnosis of mood disorders (excluding individuals with unspecified mood disorder diagnoses), outpatient status, medical stability, completion of the questionnaires and proficiency in the Korean language. The author (CHKP) reviewed the medical records of eligible patients. The study protocol received approval from the Institutional Review Board of Asan Medical Center (2024–1158), and the requirement for informed consent was exempted due to the retrospective nature of the study.

### 2.2 Measures

#### 2.2.1 Young Schema Questionnaire‐Short Form, Version 3 (YSQ‐S3)

The YSQ‐S3 is a self‐report questionnaire assessing EMSs (Young and Brown 2005). It consists of 90 items rated on a 6‐point scale (1 = “*completely untrue of me*” to 6 = “*describes me perfectly*”). These items are categorised into 18 EMSs, with higher scores indicating a stronger endorsement of dysfunctional beliefs. In our sample, most schemas demonstrated adequate internal consistency (Cronbach's *α* = 0.72 ~ 0.92), except for the entitlement/grandiosity schema (Cronbach's *α* = 0.55). The low reliability of the entitlement/grandiosity schema was consistently found in previous research (Bach et al. 2015; Hawke and Provencher 2012).

#### 2.2.2 Interpersonal Needs Questionnaire‐15 (INQ‐15)

The INQ‐15 is a self‐report measure derived from the IPTS to assess perceived burdensomeness and thwarted belongingness (Park and Kim 2019; Van Orden et al. 2010, 2012). It comprises 15 items rated on a 7‐point Likert scale (1 = “*not at all true for me*” to 7 = “*very true for me*”), divided into two factors: perceived burdensomeness (six items) and thwarted belongingness (nine items). In our sample, Cronbach's *α* values were 0.94 for perceived burdensomeness and 0.85 for thwarted belongingness, showing strong internal consistency for both factors.

#### 2.2.3 The Acquired Capability for Suicide Scale‐Fearlessness About Death (ACSS‐FAD)

Fearlessness about death was assessed using the ACSS‐FAD, a self‐administered questionnaire (Ribeiro et al. 2014; Ryu and You 2017). It comprises seven items rated on a 5‐point Likert scale (0 = “*not at all like me*” to 4 = “*very much like me*”). Higher total scores indicate a greater degree of fearlessness about death. The internal consistency was good in our study (Cronbach's *α* = 0.82).

#### 2.2.4 Depressive Symptom Inventory‐Suicide Risk Subscale (DSI‐SS)

Suicidal ideation was evaluated using the DSI‐SS, a self‐report measure developed as a part of the Hopelessness Depression Symptom Questionnaire (Metalsky and Joiner 1997; Suh et al. 2017). It comprises four items that gauge the frequency and intensity of suicidal ideation, plans, control over ideation and impulses over the past 2 weeks. The internal consistency was excellent in our sample (Cronbach's *α* = 0.93).

### 2.3 Statistical Analysis

When conducting LPA, we followed the guidelines suggested by Spurk et al. (2020) to ensure the appropriateness of the statistical method application and interpretations of the results. The determination of the number of classes followed established recommendations (Nylund et al. 2007), considering goodness‐of‐fit indices, likelihood ratios and class size, collectively emphasising parsimony. Key goodness‐of‐fit indices included the Akaike information criteria (AIC), the Bayesian information criteria (BIC) and the sample size‐adjusted BIC (SSABIC), with lower values indicating better model fit (Hu and Bentler 1999). Entropy was examined, with values closer to 1 indicating better classification (Ramaswamy et al. 1993). Profile size was also evaluated; prior research suggests avoiding profiles with < 5% of participants due to potential restrictions in power and precision (Lubke and Neale 2006). Finally, the Lo–Mendell–Rubin adjusted test (LMRA) (Lo et al. 2001) and the bootstrap likelihood ratio test (BLRT) (Nylund et al. 2007) were also performed to examine whether the model fit significantly improved when comparing a model with *k* classes to one with *k* − 1 classes. A significant *p*‐value in these tests suggests that the model with more classes has a significantly better fit.

In the second step, patients were assigned to their most likely profile based on the latent profile posterior distribution. Lastly, in the third step, suicide risk factors across the profiles were compared using the BCH (Bolck, Croon and Hagenaars) method (Asparouhov and Muthén 2014). The BCH method offers statistical advantages; it prevents potential class shifts caused by the inclusion of comparison variables and is robust to violations of normality and unequal variances across profiles (Asparouhov and Muthén 2014). Additionally, the BCH method accounts for measurement error in determining profile membership.

## 3 Results

### 3.1 LPA

**FIGURE 1:**
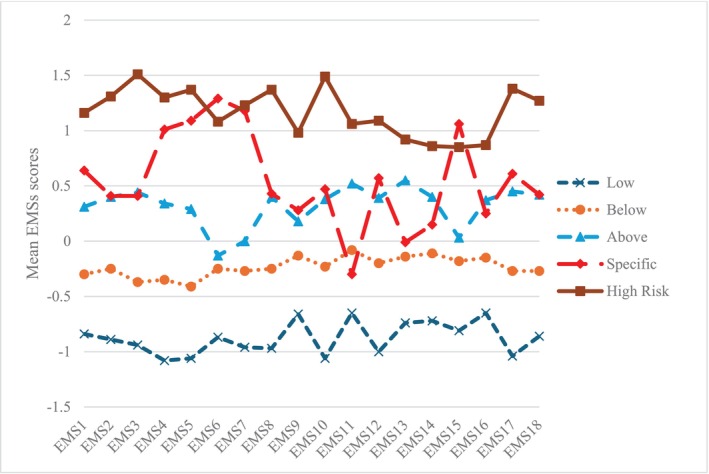
Latent profile model of EMSs in patients with mood disorders. *Note*: EMS, early maladaptive schema. Mean EMS scores were *z*‐transformed.

Five profiles yielded the optimal solution (AIC = 33013.30; BIC = 33537.84; SSABIC = 33182.18; Entropy = 0.89; smallest class = 13.99%; LMRA *p* = 0.02; BLRT *p* < 0.001) (Table 1). Figure 1 shows the mean scores for all indicators in each profile. AIC, BIC and SSABIC decreased as the number of classes increased.

**TABLE 1:** Fit indicators for two‐profile to seven‐profile solutions.

Profile	AIC	BIC	SSABIC	Entropy	LMRA(*p*)	BLRT(*p*)	Smallest class size (%)
2	35545.17	35802.75	35628.09	0.95	< 0.001	< 0.001	44.56
3	33889.20	34235.77	34000.77	0.93	< 0.001	< 0.001	24.30
4	33381.34	33816.89	33521.56	0.90	0.01	< 0.001	19.65
**5**	**33013.30**	**33537.84**	**33182.18**	**0.89**	**0.02**	**< 0.001**	**12.99**
6	32797.40	33410.92	32994.92	0.88	0.59	< 0.001	12.27
7	32610.73	33313.24	32836.90	0.89	0.16	< 0.001	4.26

*Note:* The number of profiles and model fit indices for the selected model are in bold.

Abbreviations: AIC, Akaike information criterion; BIC, Bayesian information criterion; BLRT, bootstrapped likelihood ratio test; LMRA, Lo–Mendell–Rubin adjusted test; SSABIC, sample‐size adjusted Bayesian information criterion.

Sociodemographic factors and psychiatric diagnoses among profiles are presented in Table 2. The mean scores for each profile derived from the five‐class solution are illustrated in Table 3 and Figure 1. Labels were assigned based on the mean levels of early maladaptive schema features for each class. Class 1 (19.5%), the *Low* profile, was characterised by low means for all maladaptive schemas. Class 2 (31.8%), the *Below Average* profile, was characterised by means below average for all maladaptive schemas. Class 3 (21.0%), the *Above Average* profile, was characterised by means above the sample average scores for almost all maladaptive schemas. Class 4 (13.0%), the *Specific Elevation* profile, was characterised by elevated means of social isolation/alienation, defectiveness/shame, failure, dependence/incompetence and insufficient self‐control/self‐discipline. Class 5 (14.7%), the *High Risk* profile, had the highest means for almost all maladaptive schemas.

**TABLE 2:** Sociodemographic factors and psychiatric diagnoses among profiles.

Variables	Low profile	Below average profile	Above average profile	Specific elevation profile	High risk profile
Sex (%)					
Female	97 (18.9)	169 (32.9)	105 (20.5)	65 (12.7)	77 (15.0)
Male	57 (19.9)	90 (31.5)	62 (21.7)	34 (11.9)	43 (15.0)
Age (mean)	30.55	29.65	30.04	24.19	26.23
Psychiatric diagnoses (%)					
Major depressive disorder	70 (23.7)	112 (38.0)	55 (18.6)	26 (8.8)	32 (10.8)
Bipolar I disorder	27 (21.8)	29 (23.4)	28 (22.6)	19 (15.3)	21 (16.9)
Bipolar II disorder	43 (15.1)	90 (31.7)	59 (20.8)	39 (13.7)	53 (18.7)
Other specified depressive disorder	1 (12.5)	6 (75.0)	1 (12.5)	0 (0.0)	0 (0.0)
Other specified bipolar‐related disorder	13 (14.8)	22 (25.0)	24 (27.3)	15 (17.0)	14 (15.9)

**TABLE 3:** Means of the EMSs by profile.

	EMS1	EMS2	EMS3	EMS4	EMS5	EMS6	EMS7	EMS8	EMS9	EMS10	EMS11	EMS12	EMS13	EMS14	EMS15	EMS16	EMS17	EMS18
Low profile	−0.84	−0.89	−0.94	−1.08	−1.06	−0.87	−0.96	−0.97	−0.66	−1.06	−0.65	−1.00	−0.74	−0.72	−0.81	−0.65	−1.04	−0.86
Below average profile	−0.30	−0.25	−0.37	−0.35	−0.41	−0.25	−0.27	−0.25	−0.13	−0.23	−0.08	−0.20	−0.14	−0.11	−0.18	−0.15	−0.27	−0.27
Above average profile	0.31	0.40	0.44	0.34	0.29	−0.13	−0.00	0.40	0.18	0.38	0.52	0.39	0.55	0.40	0.03	0.37	0.45	0.42
Specific elevation profile	0.64	0.41	0.41	1.01	1.09	1.29	1.18	0.43	0.28	0.47	−0.30	0.57	−0.01	0.15	1.06	0.25	0.61	0.42
High risk profile	1.16	1.31	1.51	1.30	1.37	1.08	1.23	1.37	0.98	1.49	1.06	1.09	0.92	0.86	0.85	0.87	1.38	1.27

*Note:* EMS scores were z‐transformed.

Abbreviations: EMS, early maladaptive schema; EMS1, emotional deprivation; EMS2, abandonment; EMS3, mistrust/abused; EMS4, social isolation/alienation; EMS5, defectiveness/shame; EMS6, failure; EMS7, dependence/incompetence; EMS8, vulnerability to harm or illness; EMS9, enmeshment/undeveloped self; EMS10, subjugation; EMS11, self‐sacrifice; EMS12, emotional inhibition; EMS13, unrelenting standards/hypercriticalness; EMS14, entitlement/grandiosity; EMS15, insufficient self‐control/self‐discipline; EMS16, approval‐seeking/recognition seeking; EMS17, negativity/pessimism; EMS18, punitiveness.

### 3.2 Association With Suicide Risk Factors

Suicide risk factors demonstrated significant variation across the identified profiles (Table 4). After Bonferroni correction, the *High Risk* profile exhibited significantly elevated levels of perceived burdensomeness, thwarted belongingness and suicidal ideation compared with the *Low*, *Below Average* and *Above Average* profiles. Notably, the *Specific Elevation* profile, characterised by heightened scores in social isolation/alienation, defectiveness/shame, failure, dependence/incompetence and insufficient self‐control/self‐discipline, showed comparable levels of perceived burdensomeness, thwarted belongingness and suicidal ideation to the *High Risk* profile. Additionally, the *Specific Elevation* profile showed significantly higher levels of perceived burdensomeness, thwarted belongingness and suicidal ideation compared with the *Above Average* profile, suggesting that these EMSs may confer a particularly elevated suicide risk. A clear progression in suicide risk factors was observed across the *Low*, *Below Average* and *Above Average* profiles, with each successive profile demonstrating significantly higher scores. Notably, Fearlessness about death showed no significant variation across the profiles.

**TABLE 4:** Associations between profile membership and suicide risk factors.

	Low profile	Below average profile	Above average profile	Specific elevation profile	High risk profile	*χ* ^2^	Post hoc^a^
Perceived burdensomeness	1.35	17.41	22.35	29.37	33.04	568.50***	1 < 2 < 3 < 4, 5
Thwarted belongingness	30.67	36.63	40.09	44.51	47.13	190.74***	1 < 2 < 3 < 4, 5
Fearlessness about death	15.32	15.46	15.63	17.21	16.34	5.34	—
Suicidal ideation	2.60	4.51	5.44	7.39	7.03	196.76***	1 < 2, 3 < 4, 5

*** *p* <  0.001.

^a^ After Bonferroni correction.

## 4 Discussion

To our knowledge, this is the first study to utilise a person‐centred approach to examine EMSs in a clinical sample. Our latent profile analysis identified five distinct profiles of outpatients characterised by varying levels of EMSs: *Low*, *Below Average*, *Above Average*, *Specific Elevation* and *High Risk*. These profiles demonstrated differential associations with suicide risk factors. Notably, the *Specific Elevation* profile, with significant elevations in social isolation/alienation, defectiveness/shame, failure, dependence/incompetence and insufficient self‐control/self‐discipline schemas, exhibited comparable levels of all suicide risk factors to the *High Risk* profile, which scored highest across most schemas.

Four of the elevated schemas in the *Specific Elevation* profile were consistently identified as robust predictors of suicide risk in previous studies (Dale et al. 2010; Flink et al. 2017; Khosravani et al. 2017; Nilsson 2016; Pilkington et al. 2021). This finding suggests that these EMSs may exhibit a pattern of concurrent elevation. Furthermore, it suggests that the concurrent elevation pattern may exert more unique influence on suicidal ideation relative to other schemas.

Our study found that the insufficient self‐control/self‐discipline schema was also concurrently elevated with these four EMSs in the *Specific Elevation* profile. This finding suggests that the insufficient self‐control/self‐discipline schema may also play a crucial role when co‐occurring with these schemas. Although the relationship between insufficient self‐control/self‐discipline and suicidal ideation was not clearly established in previous meta‐analyses due to the limited number of studies (Pilkington et al. 2021), other previous studies have highlighted its potential significance. It was found that insufficient self‐control, along with emotional deprivation, mistrust/abuse and social isolation, was significantly elevated in individuals who engage in self‐mutilation compared with those who did not (Castille et al. 2007). Additionally, Dale et al. (2010) identified insufficient self‐control as one of the schemas that were significantly associated with recurrent suicidal behaviour among individuals with a history of suicide attempts.

Our findings illuminate the complex interplay of EMSs in individuals with mood disorders and their relationship to suicide risk factors. The social isolation/alienation and defectiveness/shame schemas, both related to disconnection from others (Young et al. 2006), may lead individuals to perceive themselves as socially alienated and attribute this isolation to personal deficiencies. This combination of social isolation and negative self‐attribution has been shown to potentially lead to suicidal ideation (Joiner and Rudd 1995). Furthermore, the failure and dependence/incompetence schemas can be associated with perceived functional impairment (Nilsson 2016). This perception of difficulties in daily functioning may result in individuals feeling unable to fulfil expected societal roles independently, potentially contributing to suicidal ideation (Russell et al. 2009). The insufficient self‐control/self‐discipline schema relates to a perceived inability to regulate one's emotions or behaviours. Individuals experiencing difficulties in regulating their emotions may lack the capacity to tolerate psychological distress and effectively mitigate negative affect, potentially leading to suicidal ideation (Anestis et al. 2011). We observed that some individuals with mood disorders experience a combination of these distorted cognitive patterns. They may perceive themselves as inherently flawed, socially isolated, incapable of daily functioning and lacking self‐control. Notably, these individuals exhibited the highest suicide risk levels, comparable with those with elevated scores across all schemas. This study's results suggest that these five schemas may serve as optimal indicators for screening suicide risk and as potential targets for therapeutic intervention. Our findings underscore the importance of considering specific EMS profiles in assessing and treating individuals at risk for suicidal behaviour.

Contrary to expectations, fearlessness about death did not show significant differences among the EMS profiles. This may be attributed to the fact that while schemas represent an individual's distorted thinking patterns, fearlessness about death reflects a state in which the instinctive fear of death has been reduced through painful and provocative events (Van Orden et al. 2010), potentially less related to individual cognitive patterns. Moreover, although childhood abuse, known to be a major contributor to the formation of EMSs (Beck 1991; Young et al. 2006), has also been argued to increase the acquired capability for suicide (Van Orden et al. 2010), recent research has presented findings showing no significant relationship between childhood abuse and fearlessness about death (Smith et al. 2018). Recently, it has been suggested that the capability for suicide may include not only acquired capability but also dispositional capability (Klonsky and May 2015), leading to the proposal of new instruments (i.e. Suicide Capacity Scale) that complement existing instruments (Klonsky and May 2015). Future research should re‐examine the relationship between EMS profile memberships and capability for suicide measured by these new methods to determine whether EMSs are truly unrelated to capability for suicide or if the present result should be attributed to limitations in the fearlessness about death measurement tool used in this study.

The identification of schemas specifically associated with suicide risk factors has been proposed as a valuable approach in assessing suicide risk (Dutra et al. 2008). As EMSs have been linked to poor treatment outcomes (Thiel et al. 2014), the development of effective therapeutic approaches aimed at modifying these persistent maladaptive schemas emerges as a crucial component in formulating comprehensive suicide prevention strategies for patients with mood disorders. Given that the efficacy of schema therapy in reducing suicidal ideation has been supported through randomised controlled trials (Bamelis et al. 2014; Cloitre et al. 2002; Giesen‐Bloo et al. 2006), schema therapy targeting these identified schemas could be a promising option to address these EMSs and consequently mitigate suicide risk in patients with mood disorders.

The present study offers several theoretical and clinical implications. Our findings extend the literature by considering the heterogeneity in EMSs through a person‐centred approach. The results indicate that specific elevated patterns of social isolation/alienation, defectiveness/shame, failure, dependence/incompetence and insufficient self‐control/self‐discipline may exist, potentially contributing uniquely to suicide risk. Additionally, mental health professionals could be encouraged to monitor these specific schemas in patients to screen for suicide risk.

### 4.1 Limitations

First, our reliance on self‐reported measures, which are inherently subjective, represents a significant limitation of our study. The absence of neurobiological or physiological markers compromises the objectivity and depth of our understanding of EMSs. Second, the cross‐sectional design limits our ability to infer causal relationships between EMS profiles and suicide risk factors. Third, the lack of a structured interview tool for psychiatric diagnosis may call into question the accuracy of the diagnoses. Fourth, although entitlement has been observed to have a significant association with suicidal behaviour in patients with bipolar disorder (Hawke and Provencher 2012; Nilsson 2016), it did not demonstrate a specific elevated pattern alongside the five schemas in the *Specific Elevation* profile in this study. This suggests that EMS patterns may manifest differently between individuals experiencing depressive disorders and those experiencing bipolar disorders. While we were unable to conduct separate latent profile analyses for individuals diagnosed with depressive disorders (i.e. major depressive disorder) and those diagnosed with bipolar disorders due to insufficient sample size, future research could perform a multigroup latent profile analysis to investigate whether the latent profile of EMS would vary between patients with depressive disorder and bipolar disorder. Lastly, it is important to acknowledge that the profile patterns observed may not be replicated in samples with different characteristics. This could limit the broader applicability of our findings and warrants caution in their interpretation. Therefore, further validation studies with larger samples are necessary to increase the reliability of our current results. Notwithstanding these limitations, this study makes a notable contribution to the field as the first to employ latent profile analysis to examine the heterogeneity of EMSs in relation to suicide risk factors.

## 5 Conclusions

EMSs are self‐perpetuating distorted cognitive patterns that tend to persist independently of depressive symptoms and exert a negative impact on psychiatric treatment outcomes. These enduring cognitive structures may adversely influence suicidal ideation. Our latent profile analyses revealed five distinct profiles of EMSs among outpatients presenting mood symptoms: *Low*, *Below Average*, *Above Average*, *Specific Elevation* and *High Risk*. The *Specific Elevation* profiles characterised by specific elevations of social isolation/alienation, defectiveness/shame, failure, dependence/incompetence and insufficient self‐control/self‐discipline exhibited the highest levels of suicide risk factors, with no statistically significant differences from *High Risk* profiles, which demonstrated overall elevations across schemas. Our findings suggest that the five schemas exhibiting distinct elevations in the *Specific Elevation* profile may serve as valuable indicators for assessing suicide risk in clinical populations with mood disorders. These schemas also represent potential targets for therapeutic interventions aimed at mitigating suicide risk. Moreover, this study highlights the significance of acknowledging the heterogeneity within EMSs, emphasising the importance of examining unique patterns of EMSs.

## Author Contributions

**Chanhee Park**: conceptualisation, methodology, formal analysis, writing – original draft. **C. Hyung Keun Park**: conceptualisation, methodology, investigation, data curation, writing – review and editing, supervision.

## Conflicts of Interest

The authors declare no conflicts of interest.

## Data Availability

The data supporting the findings of this study are available from the corresponding author upon reasonable request.
